# Heart Disease in Patients with HIV/AIDS-An Emerging Clinical Problem

**DOI:** 10.2174/157340309788166705

**Published:** 2009-05

**Authors:** Muralikrishna Gopal, Archana Bhaskaran, Wissam I Khalife, Alejandro Barbagelata

**Affiliations:** 1 Department of Internal Medicine, University of Texas Medical Branch (UTMB), 301, University Boulevard, Galveston, TX-77550, USA; 2 Department of Internal Medicine, Division of Cardiology, University of Texas Medical Branch (UTMB), 301, University Boulevard, Galveston, TX-77550, USA

**Keywords:** HIV/AIDS, heart, cardiomyopathy, heart failure, anti-retroviral drugs, opportunistic infections.

## Abstract

HIV/AIDS (Human immunodeficiency virus/ Acquired immuno deficiency syndrome) is a growing global problem, in terms of its incidence and mortality. Patients with HIV/AIDS are living much longer with HAART (Highly active antiretroviral therapy) therapy so much so that HIV/AIDS has now become a part of the chronic disease burden, like hypertension and diabetes. Patients with HIV/AIDS and symptoms suggestive of cardiac disease represent a diagnostic and therapeutic challenge in clinical practice; Cardiologists are more frequently encountering this problem. An algorithmic, anatomic approach to diagnosis, localizing disease to the endocardium, myocardium and pericardium can be useful. An intimate knowledge of opportunistic infections affecting the heart, effects of HAART therapy and therapy for opportunistic infections on the heart is needed to be able to formulate a differential diagnosis. Effects of HAART therapy, especially protease inhibitors on lipid and glucose metabolism, and their influence on progression to premature vascular disease require consideration. Treatment of cardiac disease, in HIV/AIDS patients can vary from non-HIV patients, based on drug interactions, differences in responsiveness, and other factors; and this area requires further research.

## INTRODUCTION

The growing global epidemic of HIV/AIDS has made it the fourth leading cause of death worldwide [1]. HIV/AIDS represents a growing concern to healthcare workers and physicians worldwide with a current estimated prevalence of close to 40 million worldwide with HIV/AIDS [1]; and approx 1 in 302 or 0.33% or 1.2 million [1] people in the United States [2]. Moreover, there is not only an increasing incidence of HIV/AIDS, but also greatly improved long term survival with HAART therapy in this group of patients [3].

Incidence of opportunistic infections has decreased in the HAART era in these patients and also the metabolic effects of the Highly Active Anti-Retroviral therapy (HAART), especially the protease inhibitors (PI) become more prevalent in this patient population. Patients with HIV/AIDS are not only on HAART therapy, but also on prophylaxis for opportunistic infections, depending on their level of immunity competence and prior infections, thereby complicating the picture.

All of the above problems contribute to the increasing sub-group of patients with HIV/AIDS who have heart disease, up to 24 % in one autopsy series [4]. Heart disease in HIV/AIDS patients poses numerous diagnostic and therapeutic challenges; challenges that are unique to this population. Of particular importance to cardiologists nationwide is the increasing numbers of patients referred by internists for a cardiac work up for patients with HIV. Hence, in this review article, we aim to provide an overview of the cardiac manifestations of HIV/AIDS, including an algorithmic approach, intended for the practicing cardiologist.

This review article focuses on an anatomic rather than etiologic (see Table **1**) classification of heart disease in patients with HIV/AIDS. The three broad categories are pericardial disease, myocardial disease and endocardial disease. The fourth category includes arrhythmias, coronary artery disease, vascular disease, aneurysmal disease and pulmonary hypertension.

The etiology for each of the above type of heart disease in patients with HIV/AIDS is summarized in Table **1**.

In addition, a number of cardiac medications used for treatment of various cardiovascular conditions like arrhythmias and coronary artery disease interact with anti-retroviral therapy and an intimate knowledge of drug- drug interactions are needed in these patients (see Table **2**).

## CARDIAC DISEASE IN PATIENTS WITH HIV/AIDS

### Pericardial Disease

Diseases of the pericardium seen in patients with HIV/AIDS include pericarditis and pericardial effusions. Pericarditis in this patient population can result from bacterial pericarditis with tuberculosis being the most common, Kaposi’s sarcoma [5] or lymphomas [6]. These patients can also present with pericardial effusions, but very large effusions causing tamponade is rare. Up to 20 % of patients with AIDS have been shown to have pericardial effusions by echocardiography, and 4 % had large effusions [7]. Tuberculosis infrequently affects the pericardium, and is less common in developed countries, especially in the United States. Pericardial effusions, in patients with HIV/AIDS, even without tamponade, frequently require pericardiocentesis, since the etiology can be varied, and treatment depends on the specific etiology [8]. The only caveat of pericardiocentesis, in these patients, is the poor diagnostic yield for tuberculous pericarditis. In this setting, especially with a negative tuberculin skin test, pericardial biopsy may be more sensitive in the diagnosis. Treatment of tuberculous pericarditis requires special attention; includes anti-tuberculous therapy and corticosteroids [9, 10]. Empiric anti-tuberculous treatment should be considered in AIDS patients with undiagnosed pericardial effusions. Addition of steroids has been shown to have a significant mortality benefit in these patients [11]. And the tuberculous effusions may sometimes require pericardial fenestration. Presence of pericardial disease is a marker of poor prognosis in patients with AIDS [7].

### Myocardial Disease

Diseases of the myocardium in patients with HIV/AIDS include cardiomyopathy, myocarditis, cardiac tumors and drug toxicity. 

Left ventricular dysfunction associated with HIV/AIDS patients is most often clinically silent, and can progress to symptomatic left heart failure. The rate of progression from left ventricular dysfunction to heart failure can be considerably slowed by HAART therapy [12]. The pathogenesis of HIV- associated cardiomyopathy is exceedingly complex, and includes direct effects of the HIV virus on the heart [13], the inflammatory response of the host myocardium to the virus [14] and the presence of auto antibodies [13], as well as decreased immunity that makes them prone to infection. The myocardium lacks CD4 receptors and since this receptor is the portal of entry of HIV into the cell, the virus requires injury to the cardiac myocyte by other viruses as a pre-requisite for entry.

Myocarditis in HIV/AIDS patients is common, but identification of a specific cause can be difficult; only 20 % of myocarditis can be attributed to specific causes in HIV/AIDS patients. Causes of myocarditis can be extremely varied; and can vary from fungal-candidiasis, histoplasmosis, cryptococcosis, aspergillosis, viral-herpes simplex, cytomegalovirus, bacterial-tuberculosis or parasitic- toxoplasmosis [15]. Evaluation should include toxoplasma serology since it is a potentially treatable cause of myocarditis/cardiomyopathy in these patients. Added to the already long list is the fact that the HIV virus by itself can cause myocarditis. Drug use- cocaine and methamphetamine contributes to cardiac toxicity. Recently, selenium deficiency has been shown to be associated with cardiomyopathy [11, 16]. Reconstitution of the immune system by anti-retroviral therapy has been shown to trigger autoimmune responses that can contribute to myocardial dysfunction [17, 18].

Cardiac tumors affecting the heart in patients with HIV/AIDS are more frequently secondary than primary, as in the general population. Kaposi’s sarcoma is usually a part of disseminated mucocutaneous involvement, only rarely is the heart the sole site of involvement. Lesions are frequently asymptomatic. Lymphomas can affect the heart; lymphomas in patients with HIV/AIDS are predominantly non-Hodgkin’s [19]- they can be secondary or primary, the former being much more common than the latter. Primary Non-Hodgkin’s lymphoma originating in the heart is exceedingly rare. Primary cardiac lymphomas, usually B-cell lymphomas usually involve the right atrium, and can present with conduction disturbances, secondary to infiltration of the conduction system [20], arrythmias, superior vena cava obstruction or heart failure. Chemotherapy and radiation therapy have shown mixed results in these patients [21-23]. 

Drug toxicity can cause clinically significant myocardial dysfunction; drugs specific to the HIV/AIDS patients that can cause heart failure include zidovudine [24], interferon alpha, foscarnet, doxorubicin, pentamidine and amphotericin B.

### Endocardial Disease

Endocardial/ valvular disease in patients with HIV/AIDS can be secondary to bacterial or non-bacterial (marantic) endocarditis [4]. Bacterial endocarditis is usually secondary to intravenous drug abuse in this patient population [25], making *Staphylococcus aureus* and *Streptococcus viridans* the most common organisms and the tricuspid valve, the most common valve involved. Unlike in the myocardium, the HIV virus does not affect the endocardium directly. Non-bacterial (marantic) endocarditis is usually clinically silent, affects the tricuspid valve and can lead to embolism into the pulmonary artery, which is also clinically silent. The CD4 count has implications on the risk of developing heart disease, as well as on the prognosis. Patients with lower CD4 count, especially less than 200, have a higher risk of endocarditis, and more importantly, patients with endocarditis and lower CD4 counts have a much poorer prognosis [26]. Treatment of infective endocarditis in HIV-infected patients does not differ from those who are HIV-negative. 

### Others- Arrhythmias, Coronary Artery Disease, Vascular Disease, Aneurysmal Disease, Pulmonary Hypertension, Venous Thrombosis

#### A. Arrhythmias

Arrhythmias in patients with HIV/AIDS can be the result of drug toxicity or the secondary manifestation of myocardial disease. Pentamidine/ Pyrimethamine and TMP-SMZ (Trimethoprim- Sulfamethoxazole) used in the treatment of toxoplasmosis and PCP (Pneumocystis jirovecii) pneumonia respectively, can cause significant Q-T prolongation, and therefore torsades de pointes, that can sometimes be fatal. 29 % of hospitalized patients had QT prolongation [27] and torsades de pointes has been described in the absence of drug therapy. Ganciclovir, used in the treatment of CMV infections, can cause ventricular tachycardia. As discussed above, myocardial disease, including heart failure, and myocarditis can cause arrhythmias in patients with HIV/AIDS. Interferon alpha therapy can predispose patients to develop heart blocks and sudden cardiac death.

#### B. Coronary Artery Disease and Vascular Disease (Cerebral and Peripheral)

On one hand, although HAART therapy slows the progression to HIV- associated cardiomyopathy, HAART therapy, especially the protease inhibitors, have clinically significant effects on metabolism; causing hyperlipidemia, insulin resistance, lipodystrophy and hyperglycemia [28-30]. Different classes of HAART appear to have varying effects on the lipid profile, notably, PIs raising low density lipoproteins (LDL) [31, 32] and NNRTIs raising HDL cholesterol [31]. Accelerated atherosclerosis appears to be one of the unexpected side-effects of HAART. The relationship between anti-retroviral therapy and coronary artery disease is a topic of much debate and uncertainty. Suffice, to say, the current literature suggests that HAART therapy decreases cardiovascular risk in the short term, but prolonged use of HAART therapy, especially protease inhibitors has been shown to be associated with increased risk of CAD/ MI [28, 33-35].

Patients on HAART therapy have a 26% increased relative risk of a myocardial infarction, per year of treatment [36]. More recently, it has also been shown that NNRTIs have a low to no increased risk of myocardial infarction compared to protease inhibitors [37]. It has also been shown that Ritonavir, protease inhibitors, is associated with increase in carotid intimal wall thickness [38]. The incidence of peripheral arterial disease appears to be increased in this patient population, independent of traditional cardiovascular risk factors. Atherosclerosis and vascular disease in patients with HIV/AIDS and HAART is a topic of great interest and a complete discussion of this topic is beyond the scope of this article.

#### C. Aneurysmal Disease

Patients with HIV/AIDS are more predisposed to aneurysmal disease, especially that of the aortic and cerebral blood vessels; at a higher incidence than the general population. Aneurysms can be due to vasculitis, either by the HIV virus itself [39] or secondary infections with CMV or tuberculosis [35]. The aneurysms are usually atypical and multiple when caused by the HIV virus itself.

#### D. Pulmonary Hypertension

Patients with HIV/AIDS can develop pulmonary hypertension that is believed to be secondary to a combination of inflammation and genetic factors [40, 41]. Plexogenic arteriopathy has been described in this patient population [42, 43].

Primary pulmonary hypertension, occurs in less than 0.5% of patients with HIV infection [42], the prognosis is usually poor. Echocardiography is useful for the diagnosis of pulmonary hypertension and to rule out secondary forms. Right heart catheterization remains the gold standard for diagnosis. Histologically, plexogenic arteriopathy is found most commonly, similar to the findings in immunocompetent patients [42, 43]. Thrombotic arterial lesions and veno-occlusive disease occur far more rarely [43]. Intravenous drug users are prone to the development of pulmonary hypertension, which maybe related to intravenous injection of foreign material [44]. This pulmonary hypertension is usually worsened by poor compliance [44]. Treatment of pulmonary hypertension includes calcium-channel blockers, diuretics, anticoagulation, and prostacyclin analogues [45]. The latter, specifically epoprostenol, efficiently reduces pulmonary artery pressure both acutely and in the long term in patients with HIV infection [46]. The effect of HAART therapy on slowing the progression of pulmonary hypertension is a topic of current research. Zuber *et al. *showed improvement of pulmonary arterial pressures with long term HAART therapy [42]. Pulmonary hypertension, associated with HIV/AIDS, differs from idiopathic/ primary pulmonary hypertension in terms of rapidity of progression, is unrelated to CD4 count and is associated with a worse prognosis compared to non- AIDS patients. Bosentan/ PDE inhibitors and heart-lung transplant are usually the only treatment options that work in these sub-groups of patients [42].

#### E. Venous Thrombosis

The incidence of deep venous thrombosis has been shown to be approximately ten times more than the general population [47]. The pathogenesis of the prothrombotic state appears to be a combination of factors: increase in plasminogen activator inhibitor (PAI)- type 1, heparin cofactor II, protein S and d-dimer values [48].

## CLINICAL APPROACH TO HEART DISEASE IN HIV/AIDS PATIENTS

The history and physical examination must be used to detect symptoms and signs of cardiovascular disease in patients with HIV/AIDS. The history must include details of previous opportunistic infections, traditional risk factors for atherosclerosis, details regarding present and prior anti-retroviral therapy. One of the important questions clinicians should ask themselves is whether an HIV-positive individual is immunocompetent or immunodeficient- on the basis of a recent CD4 count and if not available, this would be necessary for further diagnostic evaluation and decisions regarding treatment and prognosis (see Fig. (**1**) for algorithm). If the patient is not already on anti-retroviral therapy and presents with cardiac symptoms, this may require referral to an infectious disease specialist for decision making regarding anti-retroviral therapy. Co-ordination of care between infectious disease and cardiology can improve the quality of care and aid in developing an individualized treatment plan based on all of the above factors. Routine use of electrocardiography or echocardiography in these patients is discouraged, especially because of the lack of evidence for finding sub-clinical disease. Shortness of breath is a common complaint, and in patients with HIV/AIDS, requires consideration of cardiomyopathy and pulmonary hypertension as possible etiologies. Transthoracic echocardiography is required for further evaluation. Drug therapy of for heart failure is not different from HIV-negative individuals, except for consideration of drug-drug interactions, especially with anti-retroviral therapy. Endomyocardial biopsy may be needed in HIV/AIDS patients with ventricular dysfunction on echocardiography to identify potentially treatable causes of myocarditis/cardiomyopathy. Lastly, cardiotoxic medications may need to be stopped in patients who have pre-existing or those who have developed significant cardiovascular disease. Management of pericardial disease, especially tuberculous effusions, is different in this patient population: addition of steroids is indicated and pericardiocentesis is needed even in the absence of tamponade. Treatment of endocarditis does not differ from HIV negative individuals. In this new era of significantly improved prognosis in patients with HIV/AIDS, both cardiac procedures and cardiovascular surgery, including valve replacement and coronary artery bypass grafting should be done in these patients, except in the setting of advanced immunosuppression or high risk of mortality from AIDS- related complications. Increased incidence of coronary artery disease, peripheral vascular disease and deep venous thrombosis has been shown in this patient population and requires careful consideration of the adverse effects of the different classes of anti-retroviral therapy.

## CONCLUSION

A patient with HIV/AIDS and symptoms suggestive of cardiac disease, a growing problem, represents a diagnostic and therapeutic challenge in clinical practice. An intimate knowledge of opportunistic infections affecting the heart, effects of HAART therapy and therapy for opportunistic infections on the heart need to be considered in the differential diagnosis. Effects of HAART therapy, especially protease inhibitors on lipid and glucose metabolism, and their influence on progression to premature vascular disease require consideration. Finally, management of these patients can vary from non-infected patients, based on drug interactions, differences in responsiveness, and other factors; and this area requires further research.

## Figures and Tables

**Fig. (1):**
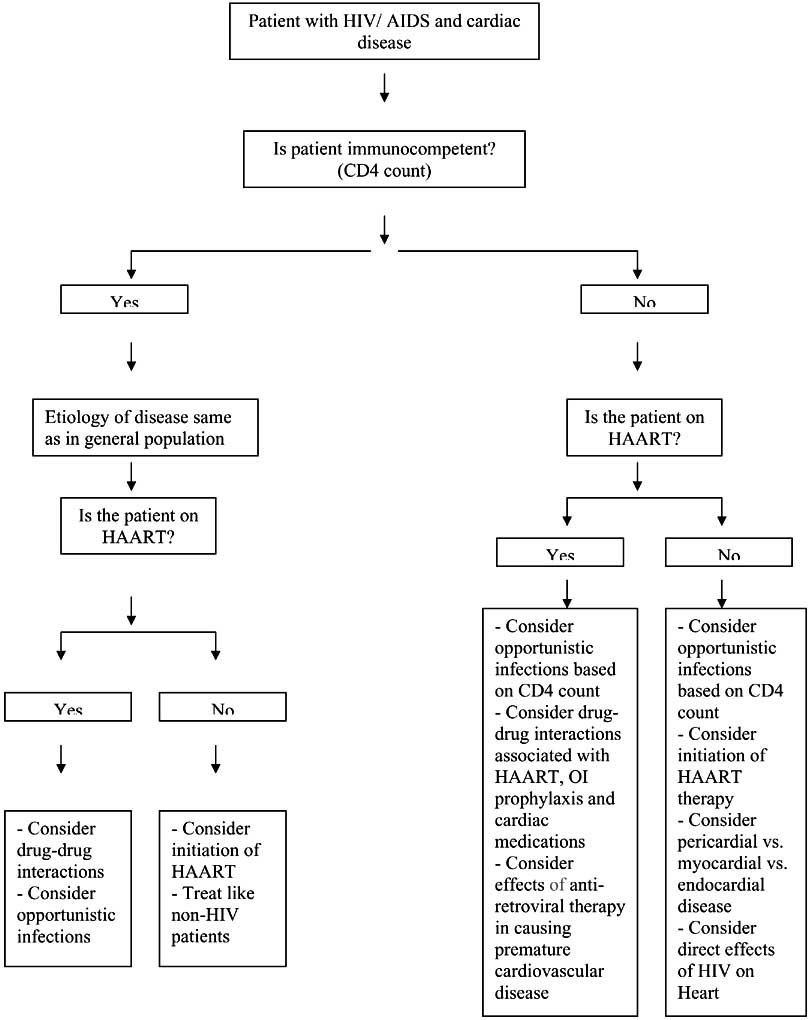
An algorithmic approach to cardiac problems in HIV/AIDS.

**Table 1:** Etiology of Cardiac Effects of HIV/AIDS

HIV directly affecting the heart
Opportunistic infections or treatment/prophylaxis of opportunistic infections
Effects of HAART on the Heart
Non-HIV cardiac risk factors (such as Diabetes Mellitus or Hypertension)
Mode of acquisition of HIV (Intravenous drug use related complications)

**Table 2:** Cardiovascular Drugs Interacting with Antiviral Therapy [49]

Cardiovascular medications that interact with anti-retrovirals
Dihydropyridine calcium-channel blockers
Sildenafil
β-Blockers, digoxin, and non-dihydropyridine calcium-channel blockers
Statins Metabolized by CYP3A4: atorvastatin, lovastatin, simvastatin, Not metabolized by CYP3A4: fluvastatin, pravastatin
Anticoagulants- warfarin, antiarrhythmics- amiodarone, antiplatelets- ASA, clopidogrel
Drugs used in HIV positive individuals that interact with cardiovascular drugs
Protease Inhibitors (PI’s)- some act as substrates, CYP enzyme inhibitor/ inducers
Nucleoside reverse transcriptase inhibitors (NRTI)-some act as substrates. CYP enzyme inhibitor/ inducers
Non-nucleotide reverse transciptase inhibitors (NNRTI)- some act as substrates. CYP enzyme inhibitor/inducers
Antibiotics- Cotrimoxazole, anti-virals- class of acyclovir, anti-fungalsazoles
Anti-tuberculous therapy
